# The passive biomechanics of human pelvic collecting lymphatic vessels

**DOI:** 10.1371/journal.pone.0183222

**Published:** 2017-08-21

**Authors:** Dimitrios Athanasiou, Lowell T. Edgar, Mohammad Jafarnejad, Katherine Nixon, Delfim Duarte, Edwin D. Hawkins, Samira Jamalian, Paula Cunnea, Cristina Lo Celso, Shunichi Kobayashi, Christina Fotopoulou, James E. Moore

**Affiliations:** 1 Department of Bioengineering, Imperial College, London, South Kensington Campus, London, United Kingdom; 2 Department of Surgery and Cancer, Ovarian Cancer Action Research Centre, Imperial College London, Hammersmith Hospital, London, United Kingdom; 3 Department of Life Sciences and the Francis Crick Institute, Imperial College London, South Kensington Campus, London, United Kingdom; 4 Immunology Division at the Walter and Eliza Hall, Institute of Medical Research, Department of Medical Biology, University of Melbourne, Victoria, Australia; 5 Department of Mechanical Engineering and Robotics, Shinshu University, Ueda, Nagano, Japan; USF Health Morsani College of Medicine, UNITED STATES

## Abstract

The lymphatic system has a major significance in the metastatic pathways in women’s cancers. Lymphatic pumping depends on both extrinsic and intrinsic mechanisms, and the mechanical behavior of lymphatic vessels regulates the function of the system. However, data on the mechanical properties and function of human lymphatics are lacking. Our aim is to characterize, for the first time, the passive biomechanical behavior of human collecting lymphatic vessels removed at pelvic lymph node dissection during primary debulking surgeries for epithelial ovarian cancer. Isolated vessels were cannulated and then pressurized at varying levels of applied axial stretch in a calcium-free Krebs buffer. Pressurized vessels were then imaged using multi-photon microscopy for collagen-elastin structural composition and fiber orientation. Both pressure-diameter and force-elongation responses were highly nonlinear, and axial stretching of the vessel served to decrease diameter at constant pressure. Pressure-diameter behavior for the human vessels is very similar to data from rat mesenteric vessels, though the human vessels were approximately 10× larger than those from rats. Multiphoton microscopy revealed the vessels to be composed of an inner layer of elastin with an outer layer of aligned collagen fibers. This is the first study that successfully described the passive biomechanical response and composition of human lymphatic vessels in patients with ovarian cancer. Future work should expand on this knowledge base with investigations of vessels from other anatomical locations, contractile behavior, and the implications on metastatic cell transport.

## Introduction

The lymphatic system plays a multifaceted role in maintaining homeostasis, being responsible for fluid and protein exchange, lipid transport, and immunity. The transport of lymph fluid is achieved through a complex network of vessels that drain around 4 liters of interstitial fluid per day whilst transferring and filtering pathogens, antigens, proteins, and lipids. Lymph enters through initial lymphatics of some tens of microns in diameter, and then is propelled through larger collecting lymphatic vessels against an unfavorable pressure gradient. The energy required for pumping is provided by contractions of mural lymphatic muscle cells (intrinsic pumping) and compression due to adjacent tissue movement (extrinsic pumping) [1]. Closely spaced one-way valves ensure antegrade flow. Disrupting the biomechanics of lymphatic transport through genetic defects, acquired conditions such as infection or cancer, or iatrogenic reasons such as surgery or radiotherapy often leads to chronic and impairing conditions such as lymphedema. However, the biomechanical aspects of lymphatic function remains an understudied subject, particularly when it comes to data on human lymphatics.

Lymphatic pumping relies on the ability of lymphatic muscle cells to contract the diameter of vessels. Experimental measurements of animal samples have shown that lymphatic vessels are more compliant than arterial or venous vessels at low pressures [2–4]. This important feature minimizes the amount of contractile pumping work lost to deforming the vessel between filling (distension) and contraction. This aids lymphatic vessels in generating significant stroke volume (proportional to the square of the change in diameter) during pumping. Intrinsic pumping is also regulated by mechanical cues such as transmural pressure and flow [1, 5–8]. Furthermore, enhanced lymph formation due to extralymphatic forces can reduce contraction rate of lymphatic muscle cells and minimize energy expenditure [1]. At higher pressures (typically greater than 3–5 cm H_2_O), these vessels experience a vast stiffness change during which further increases in pressure result in little change in diameter [3]. This increase in structural rigidity helps prevent fluid accumulation in the vessels as well as damage from overstretching. Thus, the structural characteristics of lymphatic vessels are crucial in the primary physiological function of maintaining fluid balance.

The deformation of the vessel wall due to lymphatic muscle cell contraction and the passive elastic restoring force is determined by the constitutive components of the lymphatic wall and their arrangement [3]. Recent work has shown that the rat and bovine lymphatic vessels are mainly composed of an inner layer of elastin and an outer layer of collagen fibers [3, 9, 10]. The multi-layered structure over lymphatic vessels is similar to that of small blood vessels, but lymphatic vessels have thinner walls relative to their diameter. Additionally, lymphatic muscle cells contract at frequencies of approximately 10/minute; much higher than those typical of arterial smooth muscle [11].

Most published studies on the biomechanical properties of lymphatic vessels have been restricted to animal tissue: primarily bovine, rat, and canine mesentery lymphatics from cervical and thoracic origins [2–4, 10, 12]. A notable exception is the work of Telinius et al., who studied the contractile behavior of human thoracic duct segments. Although their results provide important data on lymphatic muscle tension-diameter relationships, they did not include measurements of the passive mechanical properties [13].

Lymphogenic spread is one of the main metastatic pathways of advanced epithelial ovarian cancer, with more than 30% of the patients presenting with pelvic and/or paraaortic lymph node involvement at stages III and IV [14]. The pathways of lymphogenic metastatic routes in ovarian cancer do not commonly follow the sentinel lymph node principle, since collateral paths traverse between the right and left pelvic, paraaortic, and paracaval region. Pelvic and paraaortic lymph node dissection is often an indispensable part of extensive cytoreductive operations to achieve complete tumor clearance [15]. The availability of this tissue thus makes this disease an ideal platform to explore the architecture, biomechanics and overall properties of human collecting lymphatic vessels. Therefore, our study aims to characterize the passive mechanical properties of collecting lymphatic vessels of human pelvic origin, in normal and elongated states under different transmural pressures. Additionally, multiphoton microscopy was used to visualize and quantify the structural composition of the vessel wall.

## Materials and methods

### Lymphatic vessel isolation

**Fig 1:**
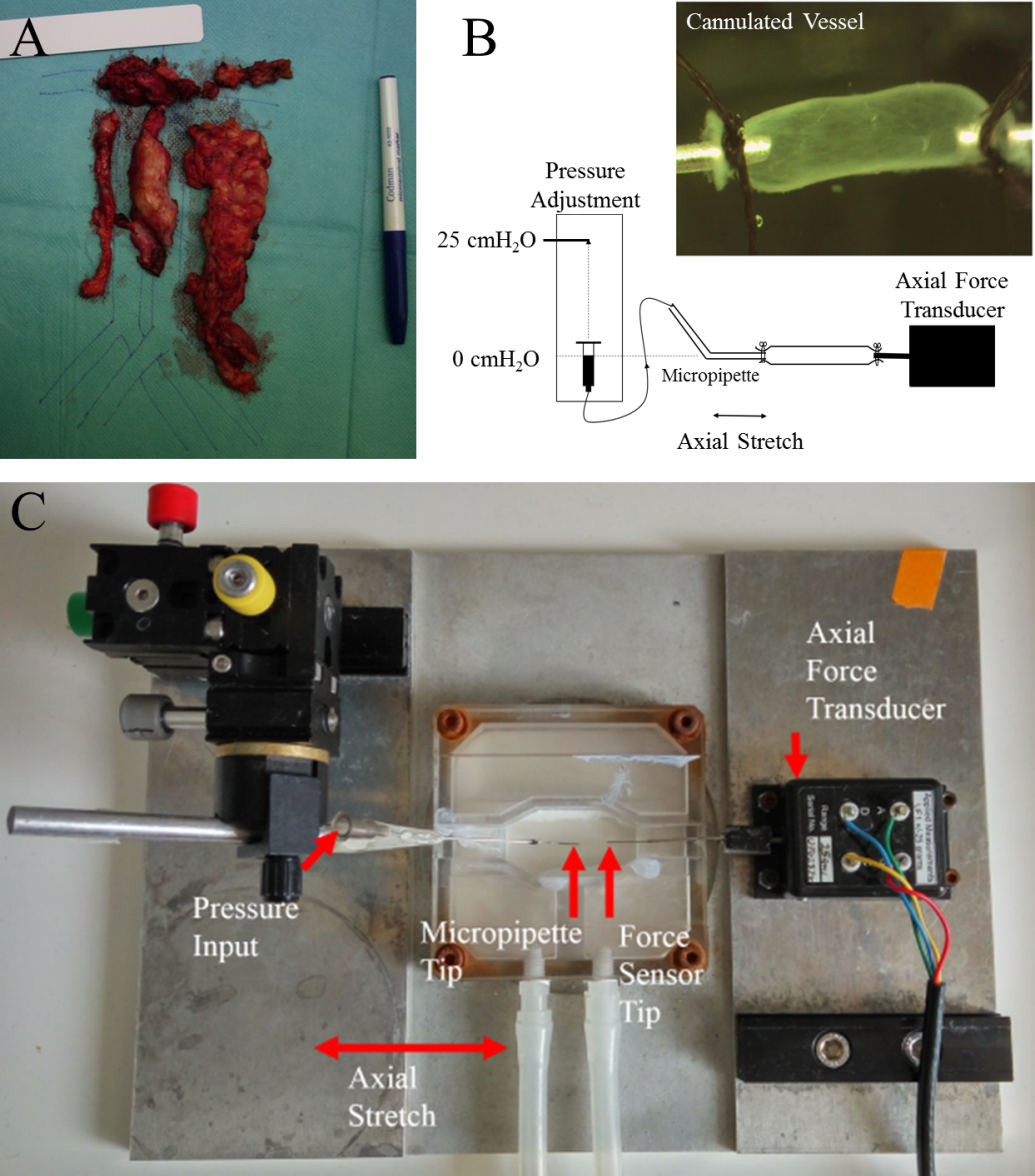
Isolation and biomechanical testing of cannulated vessels. (A) Example of lymphatic tissue excised during cytoreductive surgery along the retroperitoneal vessels. (B) A schematic of the experiments. The vessel was cannulated to a micropipette tip connected to a pressure reservoir at one end and an axial force transducer at the other. Transmural pressure was increased by adjusting the height of the pressure reservoir, and axial stretch was applied using a calibrated micrometer. An example of an image of a cannulated vessel can be seen in the inset. (C) Photograph of the cannulation chamber. This chamber was fixed on top of a stereo light microscope for imaging and measurement of vessel diameter.

Lymphatic vessels were collected from patients undergoing primary debulking surgery for advanced epithelial ovarian cancer at Hammersmith Hospital, Imperial College Healthcare NHS Trust, London, United Kingdom (Fig 1A). Informed consent was acquired from each patient. Ethics committee approval was obtained from Hammersmith and Queen Charlotte's and Chelsea Research Ethics Committee (REC reference: 05/Q0406/178), and tissue samples were provided by the Imperial College Healthcare NHS Trust Tissue Bank. As an integral part of the surgery, lymphatic vessels along the external iliac vessels were dissected together with the surrounding fibrofatty tissue as part of the lymph node dissection. No additional surgical procedures were required for harvesting of normal tissue as lymph nodes were harvested as part of the staging. Care was applied not to use any bipolar coagulation or any other thermic energy to avoid degradation of the lymphatic wall. For that reason a careful ligation of the lymph vessels with Vicyl 3–0 or PDS 4–0 was performed at the level of dissection.

Lymphatic vessels specimens were immediately submerged in cold Krebs buffer solution (4°C) with the addition of 100 unit/ml Heparin Sodium Salt after dissection. The Krebs buffer solution contained 146.9 mM NaCl, 4.7 mM KCl, 2 mM CaCl_2_, 1.2 mM MgSO_4_, 3 mM NaHCO_3_, 5 mM Na-Hepes, 1.5 mM D-glucose and the pH was adjusted to 7.4 at 37°C. All chemical components mentioned above were obtained from Sigma-Aldrich (St. Louis, MO). Lymphatic vessels of diameter 600–1200 μm were excised from the specimen and carefully isolated from the surrounding connective and fatty tissue. Six isolated vessels, each from a different patient, were cut into half (along the vessel diameter) with one half going to the group for biomechanical testing and the other half going to the group for imaging (n = 6 for each test).

### Biomechanical testing

Isolated lymphangion segments (n = 6) were transferred to a 3 mL chamber and cannulated on one end to a stainless steel micropipette and on the other end to a tip of 300 μm diameter connected to a 25 g load cell (Applied Measurements Limited, UF1) (Fig 1B). The system was mounted on a Burg-style V-track system in a Lucite fixture (Fig 1C). The vessels were visualized using a stereo light microscope (Carl Zeiss Stemi^TM^ DV4 Series Stereomicroscopes with LED Illumination) with a tablet interface (VWR VisiCam TC 10). Images of the outer diameter and the axial length of the vessels were obtained from the tablet’s image acquiring and processing software, and the axial force was measured using the software DSCUSB Toolkit. The transmural pressure was adjusted by a standing reservoir and the zero level of pressure was set using a laser level (Bulls Eye^TM^, Black&Decker). For the detection of the outer diameter a clean region in the middle of the vessel (away from boundary affects) was chosen and run through a custom edge detection macro in the image processing software FIJI [16]. The unloaded axial length was adjusted so that there was no slack in the vessel with pressure set temporarily to 7 cm H_2_O to avoid buckling. The chamber and the standing reservoir feeding the vessel with fluid were filled with a Ca^2+^-free Krebs buffer solution, having replaced CaCl_2_ with 3.0 mM EDTA, in order to inhibit lymphatic muscle cell activity and focus the study on the passive mechanical response. Diameters were measured at a single axial position and assumed to be representative of the straight lymphangion segment between valves.

Vessels were subjected to four loading cycles of transmural pressure values from 0 to 25 cm H_2_O (data taken at 0, 1, 2, 3, 4, 5, 7, 9, 11, 13, 15, 20, and 25 cm H_2_O) at different axial stretch ratios (*λ* = deformed length/unloaded length = 1.00, 1.17, 1.24, and 1.30). Use of stretch ratio is appropriate in this context of finite elasticity [17]. The *in vivo* length of the vessel was not measured. Attempting measurements of the *in vivo* axial length during human cancer surgeries would have presented significant technical challenges and ethical issues. At a fixed transmural pressure of 5 cm H_2_O, axial force versus axial length data were measured during elongation and unloading. Vessels were preconditioned prior to measurement by ramping the transmural pressure from 0 to 25 cm H_2_O five times for all length conditions stated above. Preconditioning is a common practice in soft tissue biomechanics [18] and was used in our previous study of rat vessels [3]. Note that even though we varied pressure and measured diameter, we present the results as pressure-diameter curves to be consistent with the literature in cardiovascular biomechanics. In this format, the slope of the pressure-diameter curve represents structural rigidity of the vessel, which includes both geometric and material properties. We will refer to this slope as “stiffness” henceforth.

### Pressure-diameter model

As an example of the potential applicability of these measurements, passive pressure-diameter data were fit to a model that has its origins in the collapsible tube literature. It was used in our previous lumped parameter models of lymphatic pumping [19] and also used by Rahbar et al. [3], who did similar experiments with rat mesenteric lymphatics,

$$P_{passive}\left(D\right)=P_{0}\left[\exp\left(S_{P}\left(\frac{D}{D_{0}}-1\right)\right)-0.001{\left(\frac{D}{D_{0}}\right)}^{-3}+0.05\right],$$ (Eq 1)

where *P*_0_ was the pressure scaling parameter, *S*_*P*_ is the sharpness parameter representing the shape of the transition from low stiffness to high stiffness, and *D*_*0*_ is the maximum diameter at 25 cm H_2_O. For the purposes of comparison to our previous work [3], we fit the data from the *λ* = 1.17 experiments. Based on previous experiments with isolated rat mesenteric vessels, an axial stretch of approximately 10% is required to prevent buckling at physiologic pressures. Other studies have utilized larger axial stretches [10]. We chose to normalize by the maximum diameter *D*_*0*_, similar to Rahbar et al, as projected image-based measurements of the fully pressurized diameter were more reliable those of the unpressurized diameter due to the likelihood that the vessel, near collapse, takes on a non-circular cross-section. At higher pressures, the vessels are more likely to possess a circular cross section.

### Multiphoton microscopy

Vessels were imaged within the perfusion device described above on the stage of a Zeiss LSM 780 upright confocal microscope equipped with Argon, 488, 561 and 633 nm lasers, a tunable infrared multiphoton (MP) laser (Spectraphysics Mai Tai 690–1020), 4 non-descanned detectors (NDD) and three GaAsP internal detectors. Collagen signal was detected by second harmonic generation (SHG) using MP excitation tuned to 900 nm. Elastin signal was detected using 900nm MP excitation and an NDD detector with a band pass (BP) 500–550 nm. A W Plan-Apochromat 20× DIC water immersion lens (1.0 N.A) was used to visualize the signal. Vessels were imaged in the valve and sinus regions at unloaded length and a transmural pressure of 5 cm H_2_O. Stacks were acquired with a 2.5 μm *z*-step and were ~150–200 μm in depth.

### Collagen and elastin orientation

A *z*-projection image for all the optical slices obtained from each vessel segment was created and processed in order to quantify the angular distribution of elastin and collagen fibers. Using projected images was desirable in this case because lymphatic vessel walls are very thin (<10% of their diameter, or about 100 microns for these vessels). Fiber orientation analysis was conducted using a fast-Fourier Transform (FFT) based algorithm [20]. The collagen and elastin signals were separated and subtracted from one another to account for bleed-through in the elastin channel form the strong collagen SHG signal. Histograms of frequency intensities of fiber orientation between -90° and 90° were generated based on maximum intensity *z*-projections, with 0^o^ corresponding to the axial direction (FIJI software supported by an oval profile plug-in). No directional bias was assigned to the fibers, so the histograms cover only 180°.

### Statistical analysis

A two-tailed Student’s T-test with unequal variance (Welch’s T-test) was performed on data from different groups to detect statistical difference (*α* = 0.05). We selected this test because of the large differences in standard deviation between the test cases, and used it to test for statistical significance between mean vessel diameter at axial stretch ratios of 1.00 and 1.30 at each pressure level, and between collagen and elastin fiber orientation for each fiber angle bin.

## Results

A total of six lymph vessels were isolated from six ovarian cancer patients (Fig 1A). Mean patients age was 51 years (range: 21–78 y). Disease stage ranged from FIGO IIb-IV [21]. Two of the patients had an overall positive lymph nodes status at final histology. All patients could be operated macroscopically tumor-free and were subsequently treated with systemic adjuvant platinum based chemotherapy.

**Fig 2:**
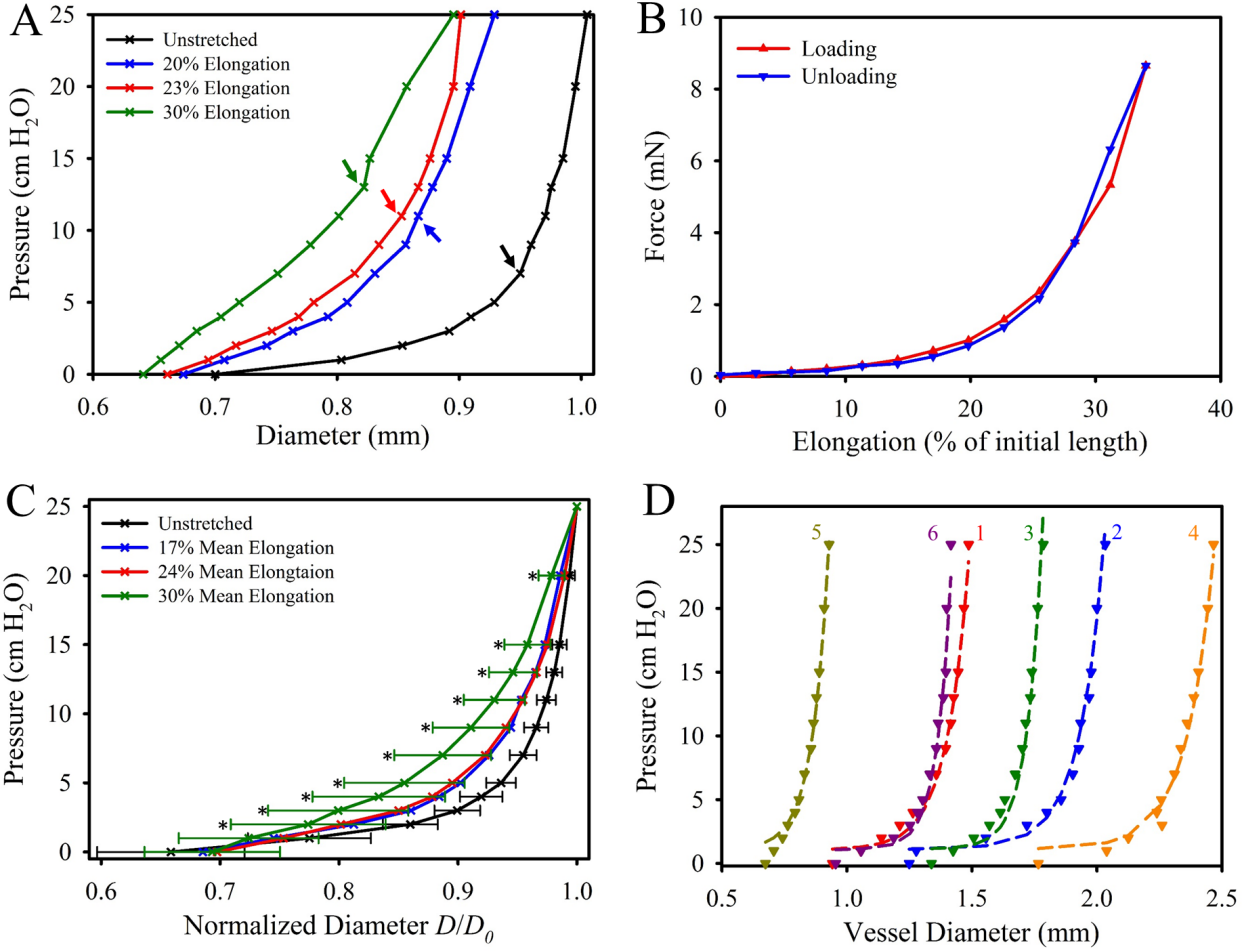
Biomechanical testing of cannulated lymphatic vessels. (A) Pressure-diameter relationship for a representative specimen at various states of elongation (0%, 20%, 23% and 30% for this specimen). The transition pressure between the low-stiffness and high-stiffness response at each elongation level is indicated by the colored arrows. (B) Force-elongation relationship for the same representative specimen at a pressure of 5 cm H_2_O. (C) The mean pressure-normalized diameter for the 6 specimens in the unstretched (black), 20% (blue), 23% (red), and 30% elongation (green) states. Error bars indicating standard deviation where only included for the 0% and 30% data for clarity (others were similar in value). An asterisk indicates a significant statistical difference detected via T-test between the 0% and 30% data for a given pressure level. (D) Pressure-diameter data from each of the six specimens (dashed lines) and the resulting fit parameters of Eq 1 as listed in Table 1 (markers).

Lymphatic vessels exhibited a highly nonlinear pressure–diameter relationship, and each vessel experienced a transition in which the rigidity (slope of the pressure-diameter curve) sharply increased (Fig 2). This transition zone from low stiffness to high stiffness occurred around 5 cm H_2_O in unstretched vessels. As the vessels were elongated, this transition became smoother and the difference in slope between the low- and high-stiffness regions decreased (Fig 2A). All specimens exhibited an elastic response during elongation, with little to no hysteresis between loading and unloading (Fig 2B). Maximum axial force during elongation ranged between 4.09 and 10.01 mN. The fully pressurized diameters of the lymphatic vessels at 0% elongation varied from 1.0 to 2.52 mm. The mean pressure-normalized diameter curve had a relatively tight distribution at 0% elongation, but the variation between specimens increased as the vessels were elongated to 30% (Fig 2C).

Pressure-diameter behavior of human lymphatic vessels was very similar to the equivalent data from rat mesentery provided by Rahbar et al., even though the human vessels were much larger, with a maximum diameter over 10× that of rat vessels. Fitting our data to the pressure-diameter equation proposed by Rahbar et al. (Eq 1) resulted in a good fit for each specimen with an mean *R*^*2*^ value of 0.989 (Table 1, Fig 2D). The pressure scaling parameter *P*_0_, which is related to the pressure value at *D*_0_, was slightly larger for the human vessels (Table 1). However, the sharpness parameter *S*_*P*_, was comparable between the human pelvic and rat mesentery vessels (Table 1), suggesting that the transition from low stiffness to high stiffness regions was similar between the two very different tissue types.

**Table 1:** Parameters fit to the model in Eq 1, originally presented by Rahbar et al. [3]. The parameters fit to each human vessel specimen in the first six columns with the mean and standard deviation of the human data in the seventh column and the mean and standard deviation of rat mesenteric vessels from Rahbar et al. [3] in the last column. Rahbar et al. tested vessel segments both upstream and downstream of a secondary lymphatic valve but found no statistical significance between the regions, so we chose to include only upstream data from their work.

	Vessel 1	Vessel 2	Vessel 3	Vessel 4	Vessel 5	Vessel 6	Mean±StdDev	Rat [3]
*P*_0_ (cm H_2_O)	23.29	24.3	24.07	23.78	24.46	22.93	23.80±0.54	18.0±0.6
*S*_*P*_	16.63	20.38	28.58	22.44	14.09	24.85	21.16±4.35	20.4±5.3
*D*_0_ (mm)	1.49	2.03	1.78	2.47	0.93	1.42	1.68±0.48	0.157±0.01
*R*^2^	0.992	0.989	0.995	0.991	0.992	0.978	0.989	—

**Fig 3:**
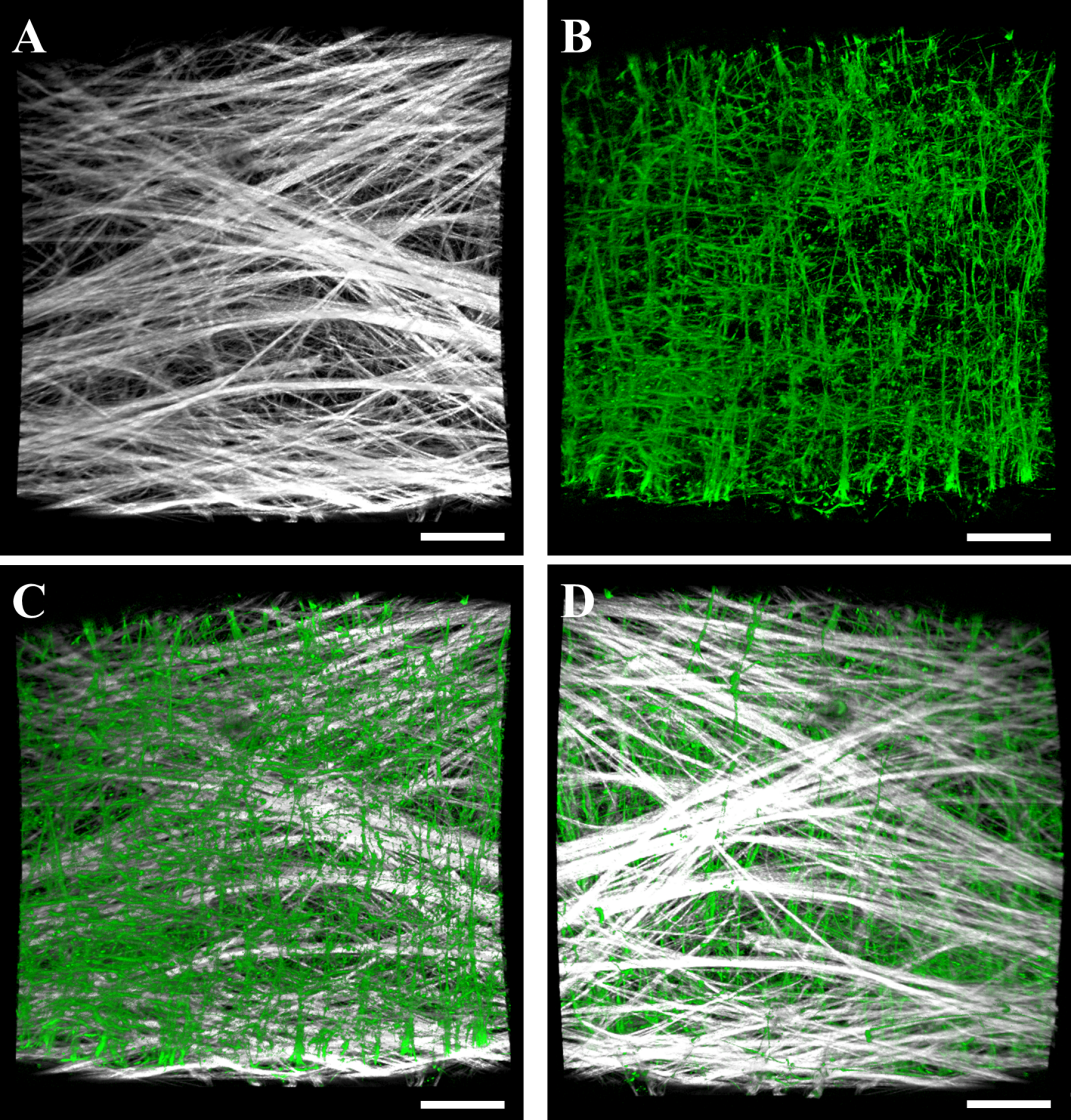
Volumetric renderings of collagen and elastin layers within in the lymphatic vessel wall imaged using multiphoton microscopy. (A) Collagen signal as viewed from the interior of the vessel. (B) Elastin signal as viewed from the interior of the vessel. The bottom panels show composite renderings of collagen (white) and elastin (green) within the interior surface of the vessel (C) and the exterior surface (D). Volumetric renderings of multiphoton image data were performed using the software FluoRender [25]. Scale bar 100 μm.

**Fig 4:**
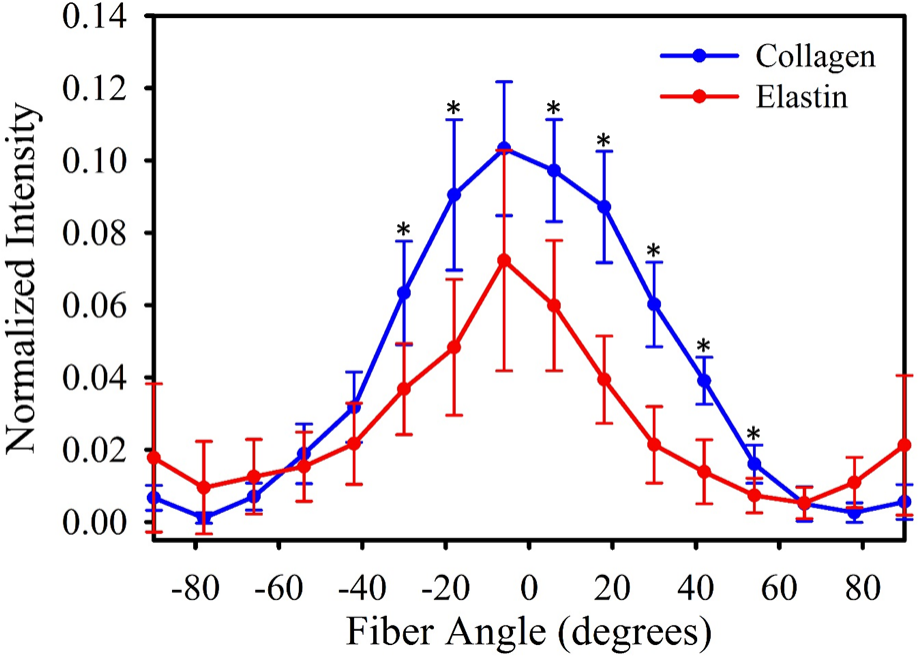
Collagen and elastin orientation as quantified by FFT. The mean collagen fibre orientation from the 6 specimens is given in blue, and the mean elastin orientation is given in red. Error bars indicate standard deviation. Orientation angles from -90° to 90°, with the axial length of the vessel orientated at 0°. Asterisks indicate a significant statistical difference between collagen and elastin orientation as detected via T-test.

Multiphoton microscopy revealed both collagen and elastin layers within the lymphatic vessel wall (Fig 3A and 3B). Collagen was found within the outer layer of the vessel wall, while elastin was found within an inner layer surrounding the lumen (Fig 4C and 4D). This layered composition was consistent along the length of the vessel. Collagen fibers were predominately oriented along the axial length of the vessel, as indicated by the distribution of fiber angles measured by FFT centered at 0° (Fig 4, blue). We found two groups of elastin bundles: the majority of bundles were aligned axially but a portion of bundles were aligned perpendicular to the axial length of the vessel along the circumference of the vessel’s interior surface, as indicated by the peaks near -90° and 90° in the elastin angle distribution (Fig 4, red).

## Discussion

Lymphogenic metastatic pathways are a major avenue of malignant disease dissemination for many important cancer types. Despite their important role with vast clinical implications in surgical approaches and systemic management of almost all cancers, only very little data exist on the basic physiological properties of human lymphatic vessels. Previous research on the pumping dynamics of animal vessels has demonstrated that the passive mechanical properties play a crucial role in generating stroke volume and subsequent lymph flow [22]. In this work, we describe and characterize the passive biomechanical properties of human lymphatic vessels for the first time using tissue sourced from lymph node dissection of disease-like epithelial advanced ovarian cancer patients (no additional procedures were required to harvest the tissue). We performed biomechanical testing of human pelvic collecting lymphatic vessels, measuring pressure-diameter and force-elongation responses. Additionally, we imaged human lymphatic vessels using optical microscopy, revealing the architecture and organization of constitutive collagen fibers and elastin bundles.

We found that cannulated human lymphatic vessels exhibited a highly nonlinear pressure-diameter relationship, similar to what has been observed in rat vessels [3, 10]. Vessels were distensible at low pressures before reaching a transition zone, after which the vessel exhibited a much stiffer response with higher pressure resulting in only minimal changes in diameter. This transition zone occurred at a higher pressure and diameter as the vessels became more elongated. These data allude to a possible tradeoff between intrinsic and extrinsic pumping in lymphatic vessels *in vivo*: as vessels become stretched due to adjacent tissue motion, lymphatic muscles cells will experience more resistance when trying to contract the vessel. However, extrinsic stretch of the vessel also contracts the diameter which may result in some fluid propulsion and pumping on its own. Caulk et al. found a similarly nonlinear pressure-diameter response in lymphatic vessels from rat thoracic ducts [10]. They found that these vessels transitioned into the high stiffness response at pressures around 2.9 cm H_2_O at 30% elongation, and this transition pressure increased to 10.4 cm H_2_O at 60% elongation. It should be noted that these rat vessels had diameters around half that of the human lymphatic vessels presented in this study.

The pressure-diameter data presented here fit well with the structural model proposed by Rahbar et al. for rat mesenteric vessels [3]. We chose our curve fit to the 17% stretch data as these data most likely best represent the nominal pre-strain vessels experience *in vivo*. However, the actual amount of stretch that lymphatic vessels experience during normal physiologic function most likely varies with both anatomical location and adjacent tissue movement. The pressure scaling parameter *P*_0_ is related to the maximum pressure used in the experiment (*P* = 1.049 *P*_0_ at *D* = *D*_0_) and was slightly higher in our results compared to the upstream vessel properties reported by Rahbar et al., as we tested our vessels to a maximum pressure of 25 cm H_2_O. The sharpness parameter *S*_*P*_, which is indicative for the sharpness of the transition from low stiffness to high stiffness, was similar between the rat and human data. These results suggest this nonlinear strain-stiffening response is common for lymphatic vessels in different species for a variety of locations (pelvic, mesenteric, thoracic), and that this response is independent of vessel size.

Multiphoton imaging revealed the vessel wall to be composed of an outer layer of axially-aligned collagen fibers and an inner layer of axially- and circumferentially-aligned elastin bundles. Elastin orientation in the lymphatic vessels was similar to elastin networks isolated from bovine aorta [23], and collagen organization was similar to that found in the inner adventitia [24]. The elastin layer determines the stiffness at low strains [23] and stores elastic energy which facilitates diameter restoration and vessel refilling after contraction, a vital component of intrinsic pumping. Axially-aligned collagen fibers within the outer layer of the vessel wall are mechanically recruited at higher levels of circumferential stretch and contribute to the transition zone and high stiffness response seen at higher pressure. The outer collagen layer creates a stiff shell around the vessel which prevents over-filling and hyper-elongation. Rat lymphatic vessels possess a similar structure, although there is some variation in fiber alignments [3, 9, 10].

There are some limitations that should be kept in mind while interpreting these data. Firstly, all specimens were taken from the same anatomical location, and it is highly likely that the structural composition/organization and subsequent pressure-diameter response of lymphatic vessels vary within the body and exhibit different behavior as one moves up the lymphatic vascular tree. Additionally, we selected vessels that appeared healthy for our experiments, but did not perform any testing to determine in the vessels were truly healthy and cancer-free. The presence of metastatic cells and cancer response within the vessels would most certainly affect their composition and biomechanical behavior. Concerning the multiphoton imaging, resolution and image quality was significantly while imaging through the thickness of the vessel walls, with image contrast suffering considerably at about 100 μm of depth. Without reliable accuracy in the *z*-direction, we were forced to perform our fiber analysis in 2D on image projections which does not quantify any alignment of laser-parallel fibers. In future work, we could perform analysis on cross-sectional vessel images or utilize varied laser power while imaging through the depth to better characterize alignment of these fibers. Lastly, we made the assumption of radial symmetry in our calculations and analysis for the sake of simplicity, but many vessels did exhibit some degree of asymmetry and it is currently unclear how this asymmetry would affect their biomechanical response. However, despite these limitations, we feel this work successfully demonstrates the passive biomechanics of human collecting lymphatics for the first time, providing a baseline for investigating the effects of disease on mechanical behavior. It is generally observed that tissues in the region of cancerous tumors are highly fibrotic, and this likely extends down to changes in the structural composition and arrangement of proteins. Our next steps will include experiments to characterize the biomechanics of cancerous lymphatic vessels along with the comparison of their properties according to the site of harvesting (i.e. pelvic, paraaortic, inguinal). As a more distant vision we could envisage biomechanical manipulation and intervention of the lymphatics to influence cancer spread and related secondary lymphedema.

## Supporting information

S1 File Raw data from vessel cannulation experiments. This spreadsheet file contains the raw data collected during the human vessel cannulation experiments. (Page 1) Diameter measurements (mm) of six specimens inflated up to a pressure of 25 cm H_2_O at various levels of vessel elongation (*λ* = 1.0, 1.17, 1.24, and 1.30). (Page 2) Pressure-diameter data at *λ* = 1.17 was fit using the structural model presented in Eq 1. This sheet contains the raw pressure-diameter data as well as the predictions of diameter given by the fit parameters in Table 1. (Page 3) Distribution of collagen and elastin fiber orientation within the vessel walls for six specimens. Orientation was measured via FFT applied to *z*-projections constructed from volumetric datasets obtained by two-photon microscopy. Data is presented as a histogram with fiber intensity given for each bin of orientation angle (degrees).
(XLSX)
